# Clinical characteristics and prognostic factors in hypertensive anterior uveitis diagnosed with polymerase chain reaction

**DOI:** 10.1038/s41598-021-87931-3

**Published:** 2021-04-23

**Authors:** Woong-Sun Yoo, Gyu-Nam Kim, Inyoung Chung, Min-Chul Cho, Yong Seop Han, Sang Soo Kang, Seung Pil Yun, Seong-Wook Seo, Seong-Jae Kim

**Affiliations:** 1 Department of Ophthalmology, Gyeongsang National University Hospital and Gyeongsang National University College of Medicine, Institute of Health Sciences, Jinju, South Korea; 2 Department of Laboratory Medicine, Gyeongsang National University Hospital and Gyeongsang National University College of Medicine, Jinju, South Korea; 3 Department of Ophthalmology, Gyeonsang National University Changwon Hospital, Changwon, South Korea; 4 Department of Anatomy and Convergence Medical Science, Institute of Health Sciences, College of Medicine, Gyeongsang National University, Jinju, South Korea; 5 Department of Pharmacology and Convergence Medical Science, Institute of Health Sciences, College of medicine, Gyeongsang National University, Jinju, South Korea

**Keywords:** Uveal diseases, Eye manifestations

## Abstract

Aim of the study is to report the clinical characteristics and prognostic factors in hypertensive anterior uveitis (AU) diagnosed with multiplex polymerase chain reaction (PCR). Eighty-eight eyes of 88 patients with hypertensive AU were enrolled from 2015 to 2019 in a tertiary center in South Korea. All patients underwent multiplex PCR that was performed using aqueous humor samples collected at first visit to detect the DNA of six herpesviruses. Twenty-eight (31.8%) eyes were PCR positive. Herpes simplex virus was found in 6 (6.8%) eyes, varicella-zoster virus in 7 (8.0%) eyes, cytomegalovirus in 14 (15.9%) eyes, and Epstein–Barr virus in 1 (1.1%) eye. On multivariate regression analysis, PCR positivity was significantly associated with coin-shaped keratic precipitates (odds ratio (OR) = 6.01, *P* = 0.044). Recurrence and final visual acuity were significantly associated with a presumed diagnosis of viral endotheliitis (OR = 21.69, *P* = 0.04 and OR = 6.3, *P* = 0.004, respectively). This study showed the importance of PCR positivity, suggesting that identification of the virus through active PCR testing could affect the course, treatment, and prognosis of hypertensive AU.

## Introduction

Anterior uveitis (AU) is the most common type in uveitis and is encountered in acute, recurrent, and chronic forms^1^. AU has various non-infective causes such as immune media or masquerade syndrome as well as infective causes such as bacterial, fungal, and viral^2^. Among the AU types, hypertensive AU is associated with rise of intraocular pressure (IOP) along with mild inflammation of the anterior chamber; a concept that was first introduced in 1948^3,4^. The herpes virus is known as a main cause of hypertensive AU, and herpes simplex virus (HSV), varicella-zoster virus (VZV), and cytomegalovirus (CMV) have been reported as causative agents for hypertensive AU in recent years^2,4^. Case reports and studies using diagnostic techniques such as polymerase chain reaction (PCR) have recently identified CMV or rubella virus as a cause of hypertensive AU, previously diagnosed as Posner–Schlossman syndrome (PSS) and Fuchs uveitis syndrome (FUS)^5,6^. Identifying the cause is very important for the treatment of hypertensive AU, and the importance of collecting aqueous humor from the anterior chamber and using PCR to detect viral DNA has been increasingly highlighted by recent studies^7–9^. However, the viral diagnostic rate using PCR is not high, and in some cases, weeks or months are required to derive the results, while at times, repeated tests are needed, complicating the diagnosis and early treatment^7,8,10^. Therefore, initial diagnosis and identification through ocular symptoms and signs are important when considering the current diagnostic techniques, and the resulting initial treatment may affect the clinical course or outcome.

However, there are few long-term and systematic studies of hypertensive AU's prevalence, causes, clinical progress, and vision outcomes. Therefore, in this study, we aimed to determine the prevalence, clinical characteristics, and progress of viral infection using multiplex PCR in patients with hypertensive AU and examine the factors affecting the PCR-positive status, recurrence, and vision outcomes, as well as obtain clinical information on hypertensive AU in Korea.

## Results

### Demographic and clinical characteristics

**Figure 1:**
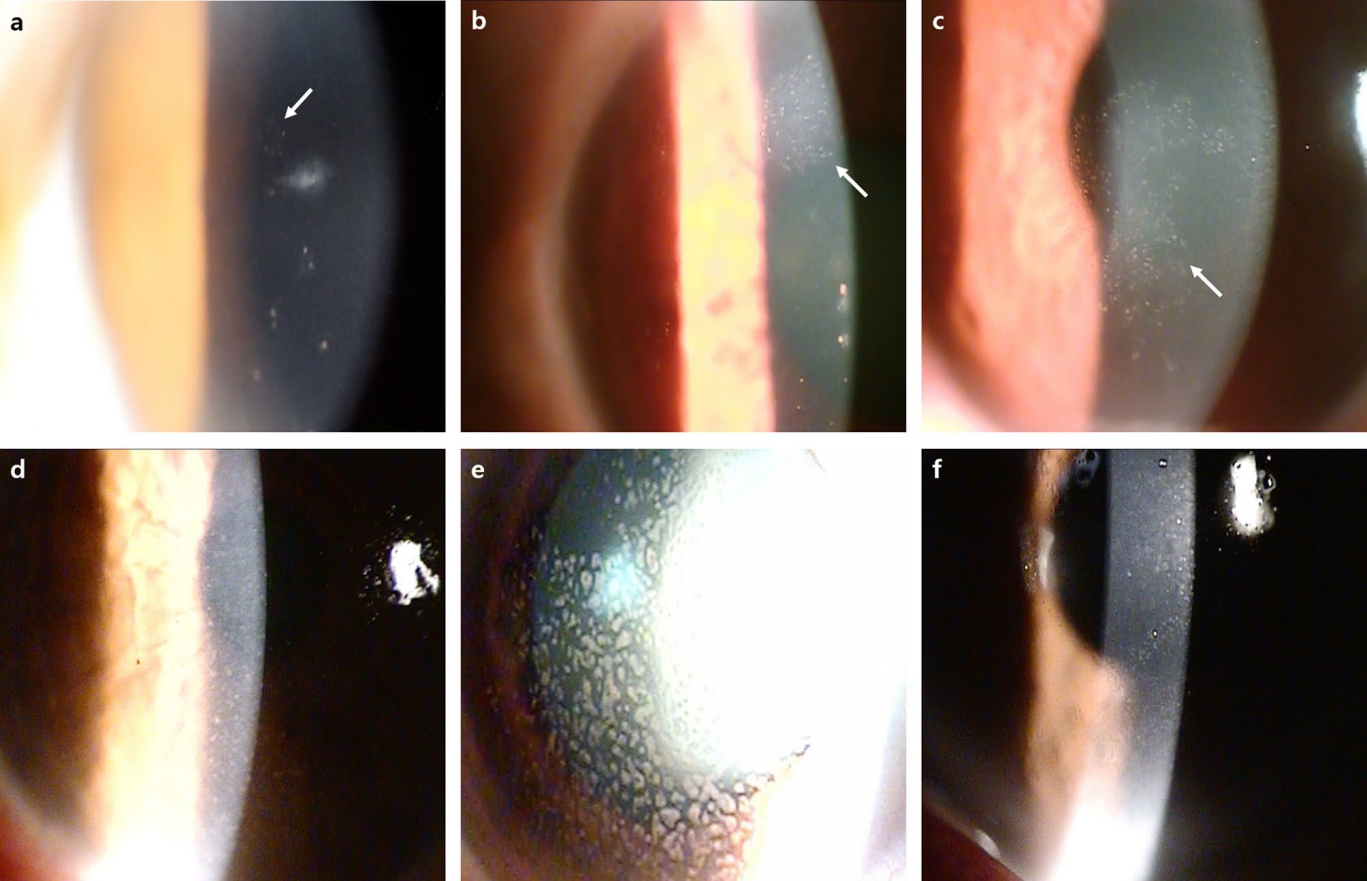
Representative anterior segment photography of patients with keratoprecipitates (KPs). (**a**–**c**) Coin-shaped KPs (white arrow) were shown which is cytomegalovirus-positive in PCR test. (**d**) Diffuse small to medium sized pigmented KPs were shown in patient with herpes simplex virus-positive. (**e**) Diffuse medium to large sized mutton-fat appearance KPs were found in patient with varicella zoster virus-positive in PCR test. (**f**) Diffuse corneal edema and multiple fine KPs were shown in patient with Epstein Barr virus-positive.

The detailed demographic and clinical characteristics of the included patients are described in Tables 1 and 2. The average follow-up period for the patients was 33.5 months. The average patient age was 61.0 ± 14.3 years, and 66 patients were male (75%); there was no difference in these characteristics between the PCR-positive and negative groups. There were 50 patients (66.8%) who used one or more anti-glaucoma medications under a diagnosis of glaucoma or ocular hypertension, and 86.4% and 51.1%, respectively, were under steroid and antiviral treatments before visiting our clinic. The use of drugs before visiting the hospital did not differ between the PCR-positive and negative groups. There were statistically significant differences according to the presumed diagnosis (*P* = 0.042) between the PCR-positive and negative groups (Table 1). Patient symptoms did not differ according to the PCR results. The median initial BCVA was 0.4 logMAR and the median initial IOP was 22.5 mmHg, which were also not significantly different between the groups. The initial central corneal thickness (CCT) did not differ between the two groups, but the initial endothelial cell count (ECC) was 2146.5/mm^2^ in the PCR-negative group and 1796.5/mm^2^ in the PCR-positive group, with the difference being significant (*P* = 0.011). Regarding the clinical features of the anterior segment, there were significant differences in the keratic precipitates (KPs), defined as fine, mutton-fat, coin-shaped (Fig. 1) and pigmented according to size and shape, between the PCR-positive and negative groups (*P* = 0.011). However, there was no difference between the two groups in terms of corneal edema, anterior chamber reaction, and iris involvement. When the glaucoma severity of the enrolled patients was analyzed, 45.5% were found to have moderate glaucoma (mean deviation from − 5.01 to − 12.00 dB in the 30-2 visual field test) or higher, which was not significantly different between the groups (Table 2).

**Table 1:** Demographic characteristics of the patients with hypertensive anterior uveitis.

	Total (N = 88)	PCR negative (N = 60)	PCR positive (N = 28)	*P*-value*
Male (%)	66 (75.0%)	43 (71.7%)	23 (82.1%)	0.428
Onset age (years) (± SD)	61.0 ± 14.3	59.8 ± 14.3	63.5 ± 14.1	0.250
Laterality (right, %)	49 (55.7%)	35 (58.3%)	14 (50.0%)	1.000
Interval between symptom onset and initial visit (days) (IQR)	20.5 (5–90)	20.5 (5–75)	22.5 (10–90)	0.553
Follow-up period (months)	33.5 (6.0–63.5)	32.0 (6.0–61.5)	38.0 (17.0–63.5)	0.172
**Previous glaucoma medication**				0.176
0	38 (43.2%)	27 (45.0%)	11 (39.3%)	
1	17 (19.3%)	12 (20.0%)	5 (17.9%)	
2	16 (18.2%)	13 (21.7%)	3 (10.7%)	
3	17 (19.3%)	8 (13.3%)	9 (32.1%)	
**Previous treatment before the visit (%)**				
Steroid	76 (86.4%)	49 (81.7%)	27 (96.4%)	0.122
Antiviral agent	45 (51.1%)	31 (51.7%)	14 (50.0%)	1.000
**Presumed diagnosis (n/%)**				0.042
Viral endotheliitis	52 (59.1%)	32 (53.3%)	20 (71.4%)	
FUS	15 (17.0%)	9 (15.0%)	6 (21.4%)	
Granulomatous AU	21 (23.9%)	19 (31.7%)	2 (7.1%)	
**Systemic disease (n/%)**				
Hypertension	29 (33.0%)	21 (35.0%)	8 (28.6%)	0.723
Diabetes	23 (26.1%)	16 (26.7%)	7 (25.0%)	1.000
Rheumatoid disease	3 (3.4%)	2 (3.3%)	1 (3.6%)	1.000

*AU *anterior uveitis, *FUS *Fuchs uveitis syndrome, *IQR *interquartile range, *PCR *polymerase chain reaction, *SD *standard deviation.

*The *P*-value was calculated between the PCR-negative and positive groups using Mann–Whitney *U* test for continuous variables and Fisher’s exact test for categorical variables.

**Table 2:** Clinical characteristics of the patients with hypertensive anterior uveitis.

	Total (N = 88)	PCR negative (N = 60)	PCR positive (N = 28)	*P*-value*
**Symptom type (n/%)**				
Ocular pain	32 (36.4%)	21 (35.0%)	11 (39.3%)	0.880
Redness	87 (98.9%)	60 (100.0%)	27 (96.4%)	0.695
Decreased visual acuity	65 (73.9%)	44 (73.3%)	21 (75.0%)	1.000
Initial BCVA (logMAR) (IQR)	0.4 (0.2–1.0)	0.4 (0.2–0.9)	0.4 (0.2–1.0)	0.888
Initial IOP (mmHg) (IQR)	22.5 (14.0–33.0)	20.5 (14.0–32.0)	26.5 (16.0–33.0)	0.358
Initial ECC (/mm^2^) (IQR)	2000.0 (1462.5–2519.0)	2146.5 (1672.5–2602.0)	1796.5 (1000.0–2154.0)	0.011
Initial CCT (um) (IQR)	556.0 (527.5–614.5)	557.5 (525.0–15.0)	545.0 (530.0–609.5)	1.000
Corneal opacity (n/%)	21 (23.9%)	17 (28.3%)	4 (14.3%)	0.241
**KPs**				0.011
Fine	44 (50.0%)	35 (58.3%)	9 (32.1%)	
Mutton-Fat	17 (19.3%)	11 (18.3%)	6 (21.4%)	
Coin-shaped	23 (26.1%)	10 (16.7%)	13 (46.4%)	
Pigmented	4 (4.5%)	4 (6.7%)	0 (0.0%)	
Corneal edema	87 (98.9%)	59 (98.3%)	28 (100.0%)	1.000
**Anterior chamber inflammation**				0.925
Trace ~ + 1	65 (73.9%)	45 (75.0%)	20 (71.4%)	
> + 1	23 (26.1%)	15 (25.0%)	8 (28.6%)	
**Iris atrophy**				0.822
Sectoral	12 (13.6%)	8 (13.3%)	4 (14.3%)	
Diffuse	10 (11.4%)	6 (10.0%)	4 (14.3%)	
**Lens status**				0.948
Pseudophakia	9 (10.2%)	6 (10.0%)	3 (10.7%)	
Phakia	79 (89.8%)	54 (90.0%)	25 (89.3%)	
Aphakia	0 (0%)	0 (0%)	0 (0%)	
**Glaucoma**				0.988
No	37 (42.0%)	25 (41.7%)	12 (42.9%)	
Early	11 (12.5%)	8 (13.3%)	3 (10.7%)	
Moderate	22 (25.0%)	15 (25.0%)	7 (25.0%)	
Advanced	18 (20.5%)	12 (20.0%)	6 (21.4%)	

*BCVA *best corrected visual acuity, *CCT *central corneal thickness, *ECC *endothelial cell count, *KP *keratoprecipitate, *IOP *intraocular pressure, *IQR *interquartile range, *MAR *minimum angle of resolution, *PCR *polymerase chain reaction.

*The *P*-value was calculated between the PCR-negative and positive groups using the Mann–Whitney *U* test for continuous variables and Fisher’s exact test for categorical variables.

### Multiplex PCR results

A total of 28/88 (31.8%) eyes were PCR positive. Among these 28 eyes, HSV was found in 6 (6.8%) eyes, VZV in 7 (8.0%) eyes, CMV in 14 (15.9%) eyes, and EBV in 1 (1.1%) eye. According to the presumed diagnosis, PCR positivity was detected in 20 eyes with viral endotheliitis, 6 eyes with FUS, and 2 eyes with granulomatous AU (Table 3).

**Table 3:** Results of multiplex PCR in hypertensive anterior uveitis.

Presumed diagnosis	Total (N = 88)	PCR negative (N = 60)	PCR positive (N = 28)				Total
			HSV	VZV	CMV	EBV	
Viral endotheliitis	52	32	6	4	10	0	20
FUS	15	9	0	2	3	1	6
Granulomatous AU	21	19	0	1	1	0	2

*AU *anterior uveitis, *CMV *cytomegalovirus, *EBV *Epstein–Barr virus, *FUS *Fuchs uveitis syndrome, *HSV *herpes simplex virus, *PCR *polymerase chain reaction, *VZV *varicella-zoster virus.

### Clinical course and outcomes

There were no significant differences between the PCR-positive and negative groups for both topical and systemic steroid treatments, but there were significant differences between the PCR-positive and negative groups for antiviral agent treatments (topical, *P* = 0.001, and systemic, *P* < 0.001). The PCR-positive group showed a significantly higher recurrence rate (*P* = 0.04) than the PCR-negative group at 53.6%, and the recurrence frequency was also significantly different between the two groups (*P* = 0.017) (Table 4).

**Table 4:** Management and recurrence during the treatment of patients with hypertensive anterior uveitis.

	Total (N = 88)	PCR negative (N = 60)	PCR positive (N = 28)	*P*-value*
**Treatment regimen**				
Topical steroid	87 (98.9%)	59 (98.3%)	28 (100.0%)	1.000
Systemic steroid	35 (39.8%)	25 (41.7%)	10 (35.7%)	0.766
Topical antiviral agent				0.001
Acyclovir	37 (42.0%)	24 (40.0%)	13 (46.4%)	
Ganciclovir	31 (35.2%)	16 (26.7%)	15 (53.6%)	
Systemic antiviral agent				< 0.001
Acyclovir	45 (51.1%)	31 (51.7%)	14 (50.0%)	
Ganciclovir	11 (12.5%)	1 (1.7%)	10 (35.7%)	
Recurrence (n/%)	32 (36.4%)	17 (28.3%)	15 (53.6%)	0.040
**Recurrence frequency**				0.017
1	11 (12.5%)	6 (10.0%)	5 (17.9%)	
2–3	17 (19.3%)	15 (25.0%)	9 (32.2%)	
> 3	8 (9%)	2 (3.3%)	6 (21.4%)	

*PCR *polymerase chain reaction.

*The *P*-value was calculated between the PCR-negative and positive groups using Mann–Whitney *U* test in continuous variables and Fisher’s exact test in categorical variables.

Best corrected visual acuity (BCVA), intraocular pressure (IOP), and CCT were not significantly different between the groups, but in the PCR-positive group, the initial ECC (1796.5/mm^3^ vs 2146.5/mm^3^; *P* = 0.011) and final ECC (1796.5/mm^3^ vs 2033.0/mm^3^; *P* = 0.04) were significantly lower than those in the PCR-negative group (Table 5).

**Table 5:** Comparison of BCVA, IOP, ECC, and CCT between the PCR-negative and PCR-positive Groups in Hypertensive Anterior Uveitis.

	Total (N = 88)	PCR negative (N = 60)	PCR positive (N = 28)	*P*-value*
**BCVA (logMAR) (IQR)**				
Initial	0.4 (0.2–1.0)	0.4 (0.2–0.9)	0.4 (0.2–1.0)	0.888
Final	0.2 (0.0– 0.7)	0.2 (0.0–0.8)	0.2 (0.0–0.6)	0.571
**IOP (mmHg) (IQR)**				
Initial	22.5 (15.0–32.0)	20.5 (14.0–32.0)	26.5 (16.0–33.0)	0.358
Final	14.0 (12.0–17.0)	14.0 (11.5–16.0)	15.5 (12.0–17.0)	0.562
**ECC (/mm**^**2**^**) (IQR)**				
Initial	2000.0 (1462.5–2519.0)	2146.5 (1672.5–2602.0)	1796.5 (1000.0–2154.0)	0.011
Final	1766.5 (936.0–2448.0)	2033.0 (1424.0–2490.5)	1282.0 (730.5–2361.5)	0.040
Unaffected eye	2536.5 (2224.5–2809.0)	2552.0 (2296.5–2871.0)	2366.0 (2195.5–2758.5)	0.241
**CCT (μm) (IQR)**				
Initial	556.0 (527.5–614.5)	557.5 (525.0–615.0)	545.0 (530.0–609.5)	1.000
Final	534.4 (481.6–587.2)	537.0 (482.9–591.1)	528.9 (478.6–579.2)	0.506
Unaffected eye	530.0 (510.0–554.0)	530.0 (510.0–553.0)	531.0 (509.5–554.0)	0.791

*BCVA *best corrected visual acuity, *CCT *central corneal thickness, *ECC *endothelial cell count, *IOP *intraocular pressure, *IQR *interquartile range, *MAR *minimum angle of resolution, *PCR *polymerase chain reaction.

*The *P*-value was calculated between the PCR-negative and positive groups using Mann–Whitney *U* test in continuous variables and Fisher’s exact test in categorical variables.

There was no difference in complications and surgery for complications between the groups. Three of 88 (3.4%) patients progressed to bullous keratopathy, and one (1.1%) patient underwent penetrating keratoplasty (Table 6).

**Table 6:** Complications and surgery for complications in patients with hypertensive anterior uveitis.

	Total (N = 88)	PCR negative (N = 60)	PCR positive (N = 28)	*P*-value*
**Complications**				0.265
Cataract	18 (20.5%)	10 (16.7%)	8 (28.6%)	
Glaucoma	39 (44.3%)	25 (41.7%)	14 (50.0%)	
Corneal perforation	2 (2.3%)	1 (1.7%)	1 (3.6%)	
Bullous keratopathy	3 (3.4%)	3 (5.0%)	0 (0.0%)	
**Surgery for complications**				0.640
Cataract surgery	13 (14.8%)	7 (11.7%)	6 (21.4%)	
Glaucoma surgery	24 (27.3%)	16 (26.7%)	8 (28.6%)	
Corneal surgery	2 (2.3%)	1 (1.7%)	1 (3.6%)	
PKP	1 (1.1%)	1 (1.7%)	0 (0.0%)	

*PCR *polymerase chain reaction, *PKP *penetrating keratoplasty.

*The *P*-value was calculated between the PCR-negative and positive groups using Fisher’s exact test.

### Risk factors for PCR positivity, recurrence, and final BCVA

**Figure 2:**
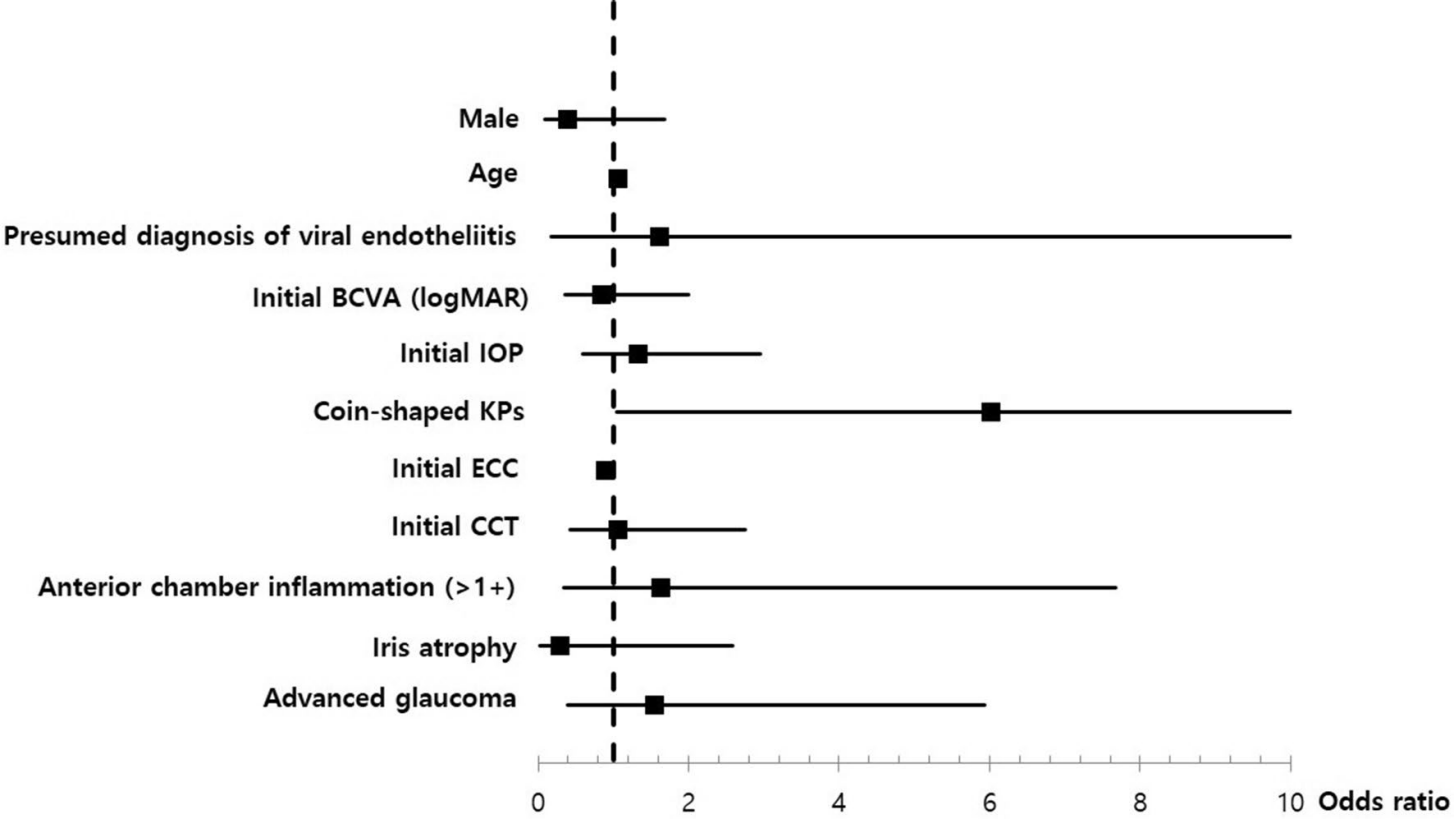
Multivariate analysis for PCR positivity in patients with hypertensive anterior uveitis. Positivity of multiplex PCR is only significantly associated with coin-shaped KPs (OR = 6.01 [95% CI 1.05–34.51], *P* = 0.044). *PCR* polymerase chain reaction, *KP* keratoprecipitate, *OR* odds ratio, *CI* confidence interval, *BCVA* best corrected visual acuity, *IOP* intraocular pressure, *ECC* endothelial cell count, *CCT* central corneal thickness.

**Figure 3:**
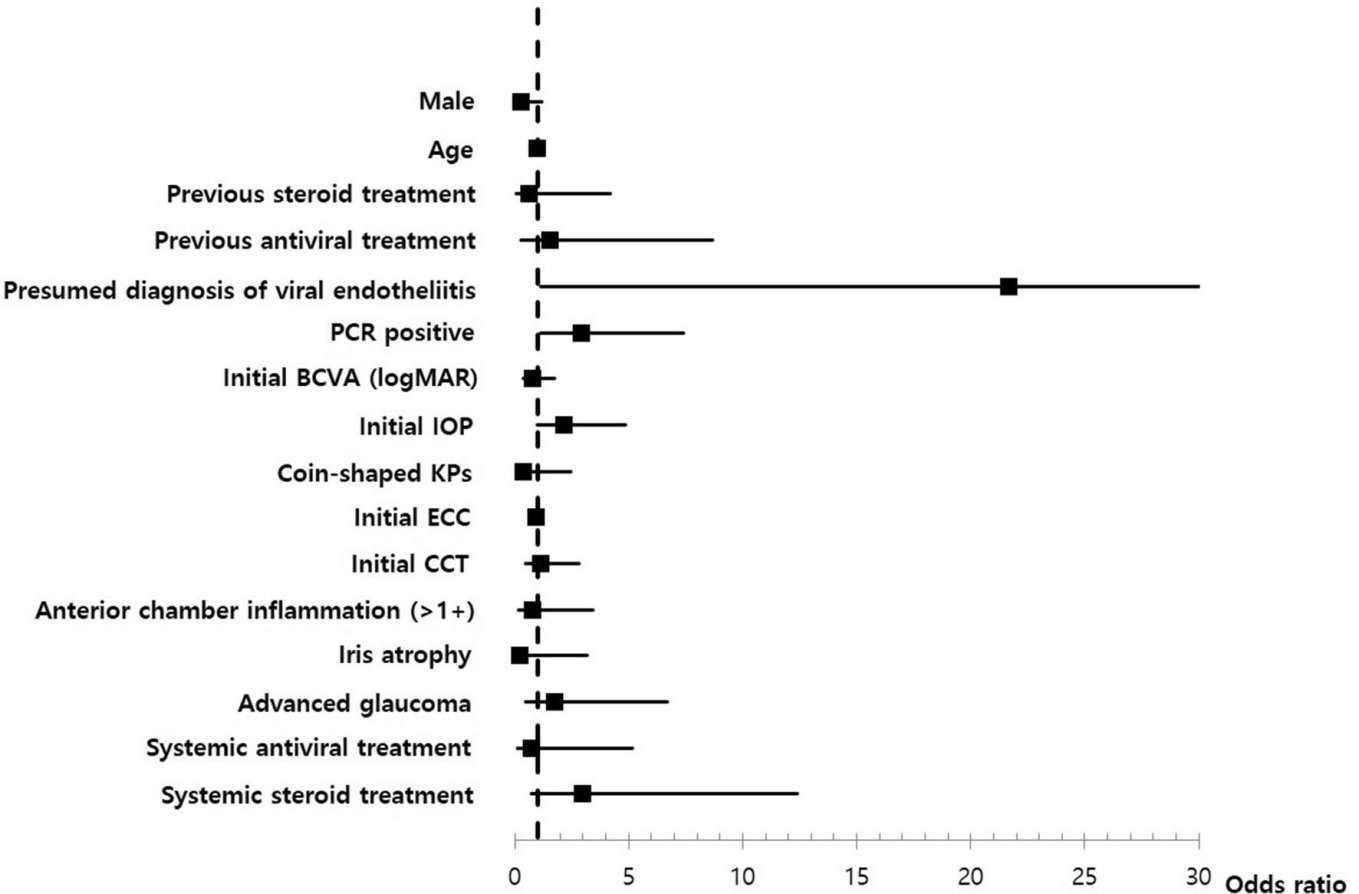
Multivariate analysis for recurrence in patients with hypertensive anterior uveitis. Recurrence was significantly associated with PCR positivity (OR = 2.92 [95% CI 1.15–7.41], *P* = 0.024) and presumed diagnosis of viral endotheliitis (OR = 21.69 [95% CI 1.14–411.53], *P* = 0.04). *PCR* polymerase chain reaction, *KP* keratoprecipitate, *OR* odds ratio, *CI* confidence interval, *BCVA* best corrected visual acuity, *IOP* intraocular pressure, *ECC* endothelial cell count, *CCT* central corneal thickness.

Multivariate regression analyses for PCR positivity, recurrence, and final BCVA were performed (see Supplementary Tables S1, S2, and S3). Higher PCR-positive rates were only observed for patients with coin-shaped KPs among multiple variables (OR = 6.01 [95% CI 1.05–34.51], *P* = 0.044) (Fig. 1). Recurrence was significantly more frequent in the PCR-positive group (OR = 2.92 [95% CI 1.15–7.41], *P* = 0.024) and in patients with a presumed diagnosis of viral endotheliitis (OR = 21.69 [95% CI 1.14–411.53], *P* = 0.04) (Fig. 2). Lower final BCVA was significantly associated with lower initial BCVA (OR = 2.43 [95% CI 1.05–5.6], *P* = 0.037) and a presumed diagnosis of viral endotheliitis (OR = 6.3 [95% CI 1.82–21.81], *P* = 0.004) (Fig. 3).

**Figure 4:**
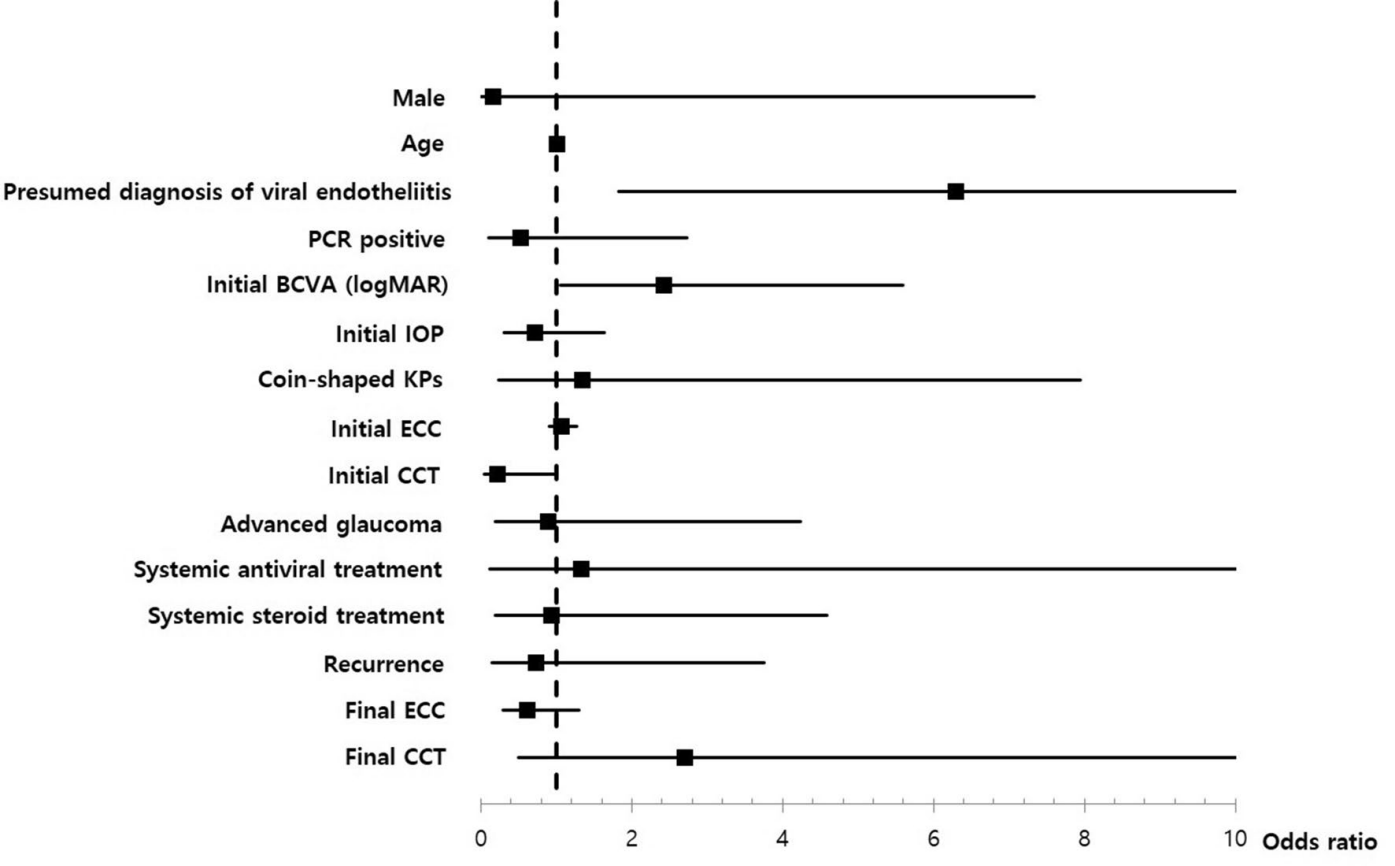
Multivariate analysis for final BCVA (logMAR) in patients with hypertensive anterior uveitis. Final BCVA is associated with low initial BCVA (logMAR) (OR = 2.43 [95% CI 1.05–5.6], *P* = 0.037) and presumed diagnosis of viral endotheliitis (OR = 6.3 [95% CI 1.82–21.81], *P* = 0.004). *PCR* polymerase chain reaction, *KP* keratoprecipitate, *OR* odds ratio, *CI* confidence interval, *BCVA* best corrected visual acuity, *IOP* intraocular pressure, *ECC* endothelial cell count, *CCT* central corneal thickness.

## Discussion

With a sample of 88 immunocompetent patients with hypertensive AU in South Korea, this study collected aqueous humor from the anterior chamber, conducted multiplex PCR on all samples, and compared and analyzed patient data according to PCR positivity. To our knowledge, this study included the largest number of immunocompetent patients with hypertensive AU among the studies conducted with Koreans^11^. Additionally, the present findings may be useful for examining the clinical course, treatment, and prognosis of hypertensive AU with a longer average follow-up period of 33.5 months than those in earlier reports.

In this study, hypertensive AU was mainly observed in middle-aged (61.0 ± 14.3 years) men (75.0%) which is similar to previous study samples in terms of age and male predominance^12,13^. In addition, PCR positivity was detected in the aqueous humor of 31.8% of enrolled patients, with CMV identified in 50% of these eyes. PCR-positive rates vary from study to study, with previous authors reporting a positive rate of approximately 38%^10^. The positive rate of viral infections in patients with hypertensive AU in Thailand from PCR of aqueous humor was 32% (10/31)^14^. And, in Belgium, among of 61 consecutive patients with hypertensive AU, 18 patients had positive in PCR (30%)^15^. In other words, the positive rate of 31.8% in this study is similar to that of Asian and European countries. CMV presents in an acute form as PSS in both Asian and Western patients, accounting for 75% and 60–93% of PSS in Singapore and Europe, respectively^12,16^. In our study, CMV was detected in 50% (14/28) of hypertensive AU patients with PCR-positive, indicating that results of this study were also not significantly different from those of other Asian countries. CMV has been reported to be a major cause of FUS in East Asia, especially in Singapore, Taiwan and Japan. Within Asia, the proportion of FUS accounted for by CMV also varies^14,17^. In our study results, CMV was detected in approximately 20% (3/15) of patients with clinical manifestations of FUS. Therefore, the PCR positive rates of HAU patients in this study, which are all Koreans, are similar to those of other countries, especially other Asian countries. In addition, CMV-positive rates have been reported to range between 22 and 34%, which is the most common causative virus among the herpes viridae family as also found in this study^4,12,18^. Characteristic coin-shaped KPs are found in hypertensive AU by CMV, which is considered to contribute to increasing the CMV-positive rates by actively testing these patients with PCR.

Virus-induced AU, which is considered a common cause of hypertensive AU, has various clinical aspects. Chan and Chee divided hypertensive AU into clinical aspects of granulomatous AU with/without corneal scar, FUS, and PSS^2^. However, the researchers classified clinical groups as “chronic AU” with granulomatous KPs, “FUS-like uveitis” with changes in the iris, such as iris atrophy, and “viral endotheliitis” accompanied by fine or coin-shaped KPs and corneal edema with inflammation of the corneal endothelium. Among the patients with hypertensive AU who underwent multiplex PCR, 59.1% had a presumed diagnosis of viral endotheliitis, 71.4% of whom were found to be PCR positive. In viral endotheliitis, 50% were CMV positive, and as noted earlier, this is considered to be the result of actively performing PCR of aqueous humor samples if characteristic coin-shaped KPs are shown.

Of all included patients, 56.8% had already been diagnosed with glaucoma before visiting our clinic; 45.5% of these patients had intermediate glaucoma and 27.3% later underwent glaucoma surgery. This clearly shows that hypertensive AU can cause vision loss due to glaucoma progression if it is not diagnosed and treated early as previously reported^19,20^. In addition, 86.4% of the treatments received before our clinic visit were steroidal, and if the treatment is not combined with antiviral agents, it is highly likely that the form of steroid responder/steroid-induced glaucoma or viral AU can worsen the progression of the disease.

Patients diagnosed and treated with hypertensive AU showed a decrease in IOP with an improvement in final BCVA. CCT had been increasing due to corneal edema prior to treatment but decreased after treatment and showed no difference from the unaffected opposite eye. However, ECC showed decrease after treatment and marked decrease compared to the other eye that was not affected. In particular, in PCR-positive patients, the decrease in initial and final ECC was significantly lower than those in PCR-negative patients, similar to previous findings, which could be due to the loss of corneal endothelial cells due to the progression of glaucoma and direct inflammation of the corneal endothelium due to the virus^10,19–21^. This reduction in ECC led to bullous keratopathy in three patients, resulting in PKP conducted in one patient.

In this study, factors that could affect PCR positivity, recurrence, and final BCVA were identified by multivariate analysis. The distribution of patients with a presumed diagnosis of viral endotheliitis, low initial ECC, and coin-shaped KPs significantly differed between the PCR-positive and negative groups. However, on multivariate analysis, only coin-shaped KPs were significant with an odds ratio of 6.01. It is therefore expected that PCR would be required for patients with coin-shaped KPs, especially considering the high detection rate of CMV and the high probability of viral endotheliitis, which may lead to rapid glaucoma progression and decrease in ECCs. In the multivariate analysis of factors related to recurrence, recurrence was associated with a presumed diagnosis of viral endotheliitis in PCR-positive patients. Especially in the case of viral endotheliitis, the odds ratio was 21.69, denoting that there was a very high risk for recurrence. In this study, CMV was detected in 50% of the 20 patients who were PCR positive and had a presumed diagnosis of viral endotheliitis, which seems to be in line with reports of frequent recurrence of CMV-associated AU in previous studies^19–22^. In the multivariate analysis of the factors influencing final BCVA, it was found that low initial BCVA and a presumed diagnosis of viral endotheliitis were associated with poor final BCVA. Patients with a presumed diagnosis of viral endotheliitis appear to show poor final BCVA due to corneal decompensation and glaucoma progression as a result of increased recurrence as seen in the sequence^20^.

This study has some limitations. First, with a retrospective research design, control of confounding variables and treatment regimens were inconsistent among patients. Second, the number of included patients was greater than those in previous studies but was still relatively low; therefore, the statistical power of the study is limited, which may have excluded otherwise meaningful factors. Third, our clinic in which this study was conducted is in a tertiary hospital with little control over previous treatment, and the point at which the aqueous humor was collected was not constant across patients with respect to the onset date. And, because patients who received topical antiviral or steroid treatment were included in this study, it cannot be completely excluded that the topical antiviral treatment can affect PCR results. Despite these limitations, this study included the largest number of Korean patients with hypertensive AU and is meaningful as a systematic inspection of factors on PCR positivity, recurrence, and final BCVA in patients with hypertensive AU with a longer than previously reported observation period.

In conclusion, this study enrolled the largest number of patients among the reported studies on hypertensive AU and provided insight into the clinical progress and outcomes of hypertensive AU with a long follow-up period. In addition, this study showed the importance of PCR positivity, suggesting that identification of the virus through active PCR testing can affect the course, treatment, and prognosis of hypertensive AU. Large-scale prospective studies to validate these findings and determine the diagnostic process and treatment regimens for hypertensive AU are needed.

## Methods

### Participants

In this study, the medical records of patients with hypertensive AU who consecutively visited the Department of Ophthalmology, Gyeongsang National University Hospital, Jinju, South Korea from January 2015 to December 2019 were retrospectively analyzed, and 88 patients (88 eyes) with hypertensive AU were included. All patients provided written consent, and the protocol of this study was approved by the Gyeongsang National University Hospital Institutional Review Boards (GNUHIRB-201711019); all procedures followed the principles of the Declaration of Helsinki.

Multiplex PCR was performed by collecting aqueous humor during the hospital visit using anterior chamber paracentesis. The patients with AU with an IOP of 25 mmHg or more were diagnosed with hypertensive AU, and patients with a history of steroid-induced ocular hypertension were excluded. Immunocompromised patients (i.e., those with a diagnosis of acquired immunodeficiency syndrome, known hematologic malignancy, or requirement for immunosuppressive drugs > 1 month) were also excluded, as well as those with inflammation of the retina. Presumed diagnosis was divided into viral endotheliitis, FUS-like AU, and granulomatous AU according to the phenotype of the clinical aspects of AU before the paracentesis based on the previously reported “Phenotypes of viral AU” by Chan and Chee in 2019^2^.

All patients underwent slit-lamp biomicroscopy, automated and/or manual refraction, CCT, ECC, Goldmann applanation tonometry, and dilated fundus examination. Pentacam (Oculus Inc., Dutenhofen, Germany) was used for CCT and the Topcon SP2000P specular microscope (Topcon, Tokyo, Japan) and CellChek XL specular microscope (Konan, Irvine, CA, USA) were used for ECC.

A comprehensive history taking of systemic or ocular pathologies was conducted for all patients. First, patients who received systemic (oral or intravenous) anti-viral or steroid treatment were excluded through the patient's history. On the other hand, patients who received topical antiviral or steroid treatment were included. Second, they were excluded in cases of suspected side effects of drugs, especially uveitis caused by anti-glaucoma medications (prostaglandin analogues or brimonidine).

To evaluate cause for anterior uveitis, all patients underwent a diagnostic workup, which included complete blood counts with erythrocyte sedimentation rate, chemistry test with C-reactive protein, serology for syphilis, toxoplasma, tuberculosis and human immunodeficiency virus, chest X-ray and urine analysis for excluding infectious caused. In addition, fluorescent antinuclear antibody test, human leukocyte antigen-B27, angiotensin-converting enzyme, c-antineutrophil cytoplasmic antibodies (ANCA), and p-ANCA was checked for systemic diseases. Patients with positive factors related to systemic disease in these tests were excluded from this study.

For grading glaucoma severity, we defined early glaucoma as a visual field defect corresponding to a mean deviation (MD) of − 5.00 dB or better, moderate glaucoma as an MD between − 5.01 and − 12.00 dB, and advance glaucoma as an MD of − 12.00 dB or worse^23^.

### Anterior chamber paracentesis

All patients underwent anterior chamber paracentesis under aseptic conditions with topical anesthesia in the supine position. After irrigating with 5% povidone and balanced salt solution, 0.15 mL of aqueous humor was collected using the 30 G needle of an insulin syringe through a clear cornea near the limbus. After sampling, we confirmed that there was no leakage from the aqueous humor and applied eyewash with moxifloxacin (Moroxacin, Hanmi Pharm., Seoul, Korea). No complications (i.e., hemorrhage, endophthalmitis, or cataract) were observed in any of the 88 patients who underwent anterior chamber paracentesis.

### Multiplex PCR

Multiplex PCR was performed as previously reported^10^. To detect viral infection in the aqueous humor, the detectable Seeplex Meningitis-V1 ACE Detection kit (v2.0; Seegene, Seoul, Korea) was used for HSV1, HSV2, VZV, EBV, CMV, and HHV6, and the results were derived through Step 3 of nucleic acid extraction, PCR, and analysis. DNA was extracted from the sample in accordance with the protocol recommended by the manufacturer (Bioneer, Seoul, Korea) using the ExiPrepViral DNA/RNA kit after the paracentesis. The extracted nucleic acid (5 μL) was mixed with the reagent (2 μL of 10 × MV1 ACE PM, 3 μL of 8-MOP solution, 10 μL of 2 × multiplex master mix) of the Seeplex Meningitis-V1 ACE Detection kit, and PCR was performed in the Veriti 96-well thermal cycler (Applied Biosystems, Waltham, MA) according to the manufacturer's instructions. The PCR conditions were as follows: (1) predenaturation at 94 °C for 15 min, (2) denaturation at 94 °C for 30 s, (3) annealing at 63 °C for 90 s, (4) 40 cycles of extension at 72 °C for 90 s, and (5) additional extension at 72 °C for 10 min. The same capacity amplification products were analyzed using 2% agarose gel electrophoresis with 0.5 mg/mL ethidium bromide staining^24^.

### Treatments

First of all, when the patient first visits the hospital, after obtaining aqueous humor for PCR test through paracentesis, topical levofloxacin (Cravit, Santen Pharmaceutical Co., Ltd., Osaka, Japan) and corticosteroid (Fumelon, Hanlim Pharm. Co. Ltd., Seoul, South Korea) was administered 6 times a day. After the PCR results were obtained, the treatment regimen was modified according to the results. Treatment regimens for CMV-positive patients included oral valganciclovir (Valcyte, Roche, Basel, Switzerland) 900 mg twice a day for 6 weeks, followed by 450 mg twice a day for 6 weeks. And also, fortified 2% topical ganciclovir (6 times/day) eye drop using intravenous ganciclovir (Cymevene, Roche, Basel, Switzerland) was started for CMV-positive AU patients during the first months, followed by 4 times/day for 2–3 months, and tapered 2–3 times/day for up to 6 months. Patients who were positive for HSV1, VZV, or EBV hypertensive AU received oral acyclovir 800 mg 3 times/day for 4–6 weeks, followed by 400–800 mg daily for 4 months. And, topical acyclovir ointment (Herpesid, Samil Co. Ltd., Seoul, South Korea) were given 4 times/day for 6 weeks and tapered to 2–3 times/day for up to 4 months. In all patients with positive results from PCR, treatment duration was at least 6 months, and topical corticosteroids and IOP-lowering agents were added according to the severity of inflammation and IOP values. In the case of patients with negative PCR results, topical corticosteroids were mainly used, and if there was no response to treatment, oral steroids (mg/kg) were used and then taped slowly. If there is no response to steroid treatment, oral acyclovir 400 mg 3 times/day (for 6–8 weeks) and topical acyclovir ointment were given 4 times/day for 6 weeks and tapered to 2–3 times/day for up to 4 months.

### Statistical analyses

Continuous variables are expressed as mean (± standard deviation; SD) and interquartile ranges, and categorical variables as numbers (percent). Continuous variables were compared between the PCR-positive and PCR-negative groups using the Mann–Whitney *U* test, and differences in categorical variables were evaluated with Fisher’s exact test. For multivariate analysis, logistic analyses were conducted, and the PCR positivity, recurrence, and risk odds ratio (95% confidence interval; CI) of final best corrected visual acuity (BCVA) were obtained. *P*-values < 0.05 were considered statistically significant. Statistical analysis was performed using R software version 2.2.1 (R Project for Statistical Computing, Vienna, Austria)^25^.

## Supplementary Information

Supplementary Information.

## Data Availability

The data used for analysis for this study are available from the corresponding author upon reasonable request.
