# Supplementary material for: Clinical characteristics and prognostic factors in hypertensive anterior uveitis diagnosed with polymerase chain reaction
Source: Sci Rep. 2021 Apr 23;11:8814. doi: 10.1038/s41598-021-87931-3 (PMC8065052; doi:10.1038/s41598-021-87931-3)
Supplement: Supplementary file 1 — Supplementary Information. [file 41598_2021_87931_MOESM1_ESM.pdf]

Clinical Characteristics and Prognostic Factors in Hypertensive Anterior Uveitis Diagnosed with Polymerase Chain Reaction

Woong-Sun Yoo[1], Gyu-Nam Kim[1], Inyoung Chung[1], Min-Chul Cho[2], Yong Seop Han[3]
Sang Soo Kang[4], Seung Pil Yun[5], Seong-Wook Seo[1*], Seong-Jae Kim[1*]

[1]Department of Ophthalmology, Gyeongsang National University Hospital and Gyeongsang National University College of Medicine, Institute of Health Sciences, Jinju, S. Korea
[2]Department of Laboratory Medicine, Gyeongsang National University Hospital and Gyeongsang National University College of Medicine, Jinju, S. Korea
[3] Department of Ophthalmology, Gyeonsang National University Changwon Hospital, Changwon, S. Korea
[4]Department of Anatomy and Convergence Medical Science, Institute of Health Sciences, College of Medicine, Gyeongsang National University, Jinju, S. Korea
[5]Department of Pharmacology and Convergence Medical Science, Institute of Health Sciences, College of medicine, Gyeongsang National University, Jinju, S. Korea

Supplementary Table S1. Multivariate Analysis for PCR Positivity in Patients with Hypertensive Anterior Uveitis

	Adjusted OR (95% CI)	*P*-value*
Male sex	0.39 (0.09–1.68)	0.350
Age	1.06 (1–1.13)	0.052
Presumed diagnosis of viral endothelitis	1.61 (0.17–15.64)	0.680
Initial BCVA (logMAR)	0.84 (0.36–2)	0.698
Initial IOP	1.33 (0.6–2.96)	0.483
Coin-shaped KPs	6.01 (1.05–34.51)	0.044
Initial ECC	0.9 (0.8–1.01)	0.069
Initial CCT	1.07 (0.42–2.76)	0.888
Anterior chamber inflammation (> 1+)	1.63 (0.34–7.67)	0.539
Iris atrophy	0.29 (0.03–2.58)	0.267
Advanced glaucoma	1.54 (0.4–5.94)	0.530

BCVA = best corrected visual acuity; CCT = central corneal thickness; CI = confidence interval; ECC = endothelial cell count; IOP = intraocular pressure; KP = keratoprecipitate; MAR = minimum angle of resolution; OR = odds ratio

* The *P*-value was calculated with Wald's test

Supplementary Table S2. Multivariate Analysis for Recurrence in Patients with Hypertensive Anterior Uveitis

	Adjusted OR (95% CI)	*P*-value*
Male sex	0.28 (0.06–1.22)	0.090
Age	1.0044 (0.9501–1.0617)	0.878
Previous steroid treatment	0.63 (0.09–4.21)	0.629
Previous antiviral treatment	1.53 (0.27–8.66)	0.632
Presumed diagnosis of viral endothelitis	21.69 (1.14–411.53)	0.040
PCR positive	2.92 (1.15–7.41)	0.024
Initial BCVA (logMAR)	0.81 (0.37–1.77)	0.601
Initial IOP	2.17 (0.97–4.84)	0.059
Coin-shaped KPs	0.38 (0.06–2.46)	0.313
Initial ECC	0.93 (0.84–1.04)	0.201
Initial CCT	1.16 (0.48–2.82)	0.746
Anterior inflammation (> 1+)	0.77 (0.17–3.44)	0.539
Iris atrophy	0.24 (0.02–3.18)	0.278
Advanced glaucoma	1.77 (0.47–6.67)	0.400
Systemic antiviral treatment	0.75 (0.11–5.16)	0.767
Systemic steroid treatment	3.00 (0.73–12.38)	0.129

BCVA = best corrected visual acuity; CCT = central corneal thickness; CI = confidence interval; ECC = endothelial cell count; IOP = intraocular pressure; KP = keratoprecipitate; MAR = minimum angle of resolution; OR = odds ratio; PCR = polymerase chain reaction

* The *P*-value was calculated with Wald's test

Supplementary Table S3. Multivariate Analysis for Final BCVA (logMAR) in Patients with Hypertensive Anterior Uveitis

	Adjusted OR (95% CI)	*P*-value*
Male sex	0.16 (0–7.34)	0.350
Age	1.0096 (0.9609–1.0608)	0.705
Presumed diagnosis of viral endothelitis	6.3 (1.82–21.81)	0.004
PCR positive	0.52 (0.1–2.73)	0.436
Initial BCVA (logMAR)	2.43 (1.05–5.6)	0.037
Initial IOP	0.71 (0.31–1.63)	0.423
Coin-shaped KPs	1.35 (0.23–7.94)	0.737
Initial ECC	1.07 (0.91–1.27)	0.408
Initial CCT	0.22 (0.05–1.01)	0.052
Advanced glaucoma	0.89 (0.19–4.23)	0.881
Systemic antiviral treatment	1.33 (0.12–14.37)	0.814
Systemic steroid treatment	0.93 (0.19–4.59)	0.933
Recurrence	0.73 (0.14–3.76)	0.705
Final ECC	0.62 (0.29–1.3)	0.204
Final CCT	2.7 (0.5–14.44)	0.246

BCVA = best corrected visual acuity; CCT = central corneal thickness; CI = confidence interval; ECC = endothelial cell count; IOP = intraocular pressure; KP = keratoprecipitate; MAR = minimum angle of resolution; OR = odds ratio; PCR = polymerase chain reaction

* The *P*-value was calculated with Wald's test
